# Host clustering of Campylobacter species and enteric pathogens in a longitudinal cohort of infants, family members and livestock in rural Eastern Ethiopia

**DOI:** 10.1186/s40168-025-02203-w

**Published:** 2025-11-03

**Authors:** Zelalem Mekuria, Loic Deblais, Amanda Ojeda, Bahar Mummed, Nitya Singh, Wondwossen Gebreyes, Arie H. Havelaar, Gireesh Rajashekara

**Affiliations:** 1 College of Veterinary Medicine, Veterinary Preventive Medicine, The Ohio State University, Columbus, OH USA; 2 Global One Health Initiative (GOHi), Office of International Affairs, The Ohio State University, Columbus, OH USA; 3 Center for Food Animal Health, The Ohio Agricultural Research and Development Center, College of Food, Agriculture and Environmental Sciences, The Ohio State University, Wooster, OH USA; 4 Hypercell Technologies, Ithaca, NY USA; 5 Emerging Pathogens Institute, Department of Animal Sciences, Global Food Systems Institute, University of Florida, Gainesville, FL USA; 6 Department of Microbiology and Cell Science, Institute of Food and Agricultural Sciences, University of Florida, Gainesville, FL USA; 7 College of Veterinary Medicine, Haramaya University, Dire Dawa, Ethiopia; 8 College of Veterinary Medicine, University of Illinois, Urbana, IL USA

**Keywords:** *Campylobacter*, Enteric pathogens, Shotgun metagenomics, Eastern Ethiopia, Longitudinal cohort, Human, Livestock

## Abstract

**Background:**

Livestock are recognized as major reservoirs for *Campylobacter* species and other enteric pathogens, posing infection risks to humans. High prevalence of *Campylobacter* during early childhood has been linked to environmental enteric dysfunction and stunting, particularly in low-resource settings.

**Methods:**

A total of 280 samples from *Campylobacter* positive households with complete metadata were analyzed by shotgun metagenomic sequencing followed by bioinformatic analysis via the CZ-ID metagenomic pipeline (Illumina mNGS Pipeline v7.1). Further statistical analyses in JMP PRO 16 explored the microbiome, emphasizing *Campylobacter* and other enteric pathogens. Two-way hierarchical clustering and split k-mer analysis examined host structuring, patterns of co-infections and genetic relationships. Principal component analysis was used to characterize microbiome composition across the seven sample types.

**Results:**

The study identified that microbiome composition was strongly host-driven, with more than 3844 genera detected, and two principal components explaining 62% of the total variation. Twenty-one dominant (based on relative abundance) *Campylobacter* species showed distinct clustering patterns for humans, ruminants, and broad hosts. The broad-host cluster included the most prevalent species, *C*. *jejuni*, *C*. *concisus*, and *C*. *coli*, present across sample types and a sub-cluster within *C*. *jejuni* involving humans, chickens, and ruminants. *Campylobacter* species from chickens showed strong positive correlations with mothers (*r* = 0.76), siblings (*r* = 0.61) and infants (*r* = 0.54), while co-occurrence analysis found a higher likelihood (Pr > 0.5) of pairs such as *C*. *jejuni* with* C*. *coli*, *C*. *concisus*, and* C*. *showae*. Analysis of the top 50 most abundant microbial taxa showed a distinct cluster uniquely present in human stool and absent in all livestock. The study also found frequent co-occurrence of *C*. *jejuni* with other enteric pathogens such as *Salmonella*, and *Shigella*, particularly in human and chicken. Additionally, instances of *Candidatus* *Campylobacter infans* (*C*. *infans*) were identified co-occurring with *Salmonella* and *Shigella* species in stool samples from infants, mothers, and siblings.

**Conclusions:**

A comprehensive analysis of *Campylobacter* diversity in humans and livestock in a low-resource setting revealed that infants can be exposed to multiple *Campylobacter* species early in life. *C*. *jejuni* is the dominant species with a propensity for co-occurrence with other notable enteric bacterial pathogens, including *Salmonella,* and *Shigella*, especially among infants.

Video Abstract

**Supplementary Information:**

The online version contains supplementary material available at 10.1186/s40168-025-02203-w.

## Introduction

Enteric infections caused by bacterial, viral, or parasitic infections are the second leading cause of mortality globally, after lower respiratory tract infections, among children younger than 5 years [1]. Through foodborne, waterborne, zoonotic, or community-acquired route, enteric infections exert a disproportionate toll on children in low- and middle-income countries (LMICs), where poor sanitation, close contact with animals, and limited access to healthcare create conditions for frequent and recurrent exposure [2]. The World Health Organization (WHO) estimates that contaminated food leads to 600 million illnesses and more than 400,000 deaths annually [3, 4], with nearly one-third affecting children’s in LMIC, contributing to malnutrition, stunting, and long-term cognitive deficits [3, 5, 6]. In addition to the human toll, the economic impact of enteric infections is substantial, resulting in an estimated total productivity loss in LMIC of US$95 billion per year, with an additional predicted annual cost of treating these infections reaching US$15 billion [7, 8].

In Ethiopia, diarrheal disease remains a leading cause of child mortality, exacerbated by inadequate access to clean water, sanitation, and hygiene services [9, 10]. The WHO estimates foodborne pathogen-associated deaths per 100,000 population in Ethiopia and diarrhea is the second most prevalent cause of infants mortality [11]. Bacterial agents include *Campylobacter* species, diarrheagenic *Escherichia coli*, non-typhoidal *Salmonella* *enterica*, *Shigella* species, while viral agents include rotavirus and norovirus, are commonly implicated particularly among children under five [5]. Importantly, persistent enteric infections with organisms like *Campylobacter* are increasingly recognized not only for their acute effects but also for their role in environmental enteric dysfunction (EED) and subsequent child malnutrition and stunting [12, 13].

While *Campylobacter jejuni* and *Campylobacter coli* are the most well-studied species globally, accounting for the majority of culture-confirmed human campylobacteriosis [2], recent studies suggest that these species represent a fraction of the broader *Campylobacter* burden in LMICs [14, 15]. Using molecular and metagenomic analyses, studies have uncovered broader diversity among *Campylobacter* species in children and animal reservoirs, with frequent detection of non-*C*. *jejuni*/*coli* species such as *C*. *concisus*, *C*. *upsaliensis*, *C*. *fetus*, and novel taxa like “*Candidatus* Campylobacter infans” [14–16]. These species have often gone undetected due to diagnostic limitations, including reliance on culture-based or targeted molecular assays that preferentially detect *C*. *jejuni and C*. *coli*. Consequently, the true prevalence, ecological distribution, and clinical relevance of other *Campylobacter* spp. remain poorly characterized, particularly in high-risk settings such as Ethiopia, where human-livestock cohabitation is common and exposure to zoonotic pathogens is high [17].

Our previous investigations, conducted in Haramaya district (in five kebeles), Eastern Ethiopia, using metatranscriptomic sequencing, demonstrated high prevalence of *Campylobacter* in young children. The study also assessed the diversity of *Campylobacter* species, and interactions with child health, specifically EED and stunting [15]. Building upon these findings, our team embarked on a broader study spanning from December 2020 to June 2022 [18]. This longitudinal investigation included stool samples (from infants, mothers, and siblings), livestock feces (from cattle, chickens, goats, and sheep), and the environment (*n* = 1644). The result showed potential routes for *Campylobacter* transmission in eastern Ethiopia and a correlation in the detection of *Campylobacter* species between infants and mothers and environmental sources [19]. Despite this growing body of evidence, a significant knowledge gap still persists on understanding the diversity and co-circulation of *Campylobacter* species at the human-livestock interface. In particular, there is limited data on the prevalence of non-*C*. *jejuni*/*coli* species in livestock and humans sharing the same environment, and their role in co-infections, or zoonotic transmission cycles. Many of these species are difficult to culture and shotgun metagenomics sequencing has been shown to accurately identify *Campylobacter* diversity at the species level, even in instances of low levels of genetic material in the samples [15, 16, 20, 21].

The objectives of this study were to use shotgun metagenomic sequencing to:

1. Identify diversity of *Campylobacter* species in a birth cohort of infants in a rural region in Eastern Ethiopia at four different time points in their first year of life.
2. Compare the *Campylobacter* species diversity in infants with other humans (siblings, mothers) in their households and livestock (cattle, goats, sheep and chickens) in their homestead.
3. Identify co-circulation of other enteric pathogens of significance for infant health.

## Materials and methods

### Study area and design

A longitudinal study involving 106 infants (ages ranging from 7 days to 13 months) was conducted from December 2020 to June 2022. Participants were selected randomly from 10 rural kebeles (synonymous with villages) of Haramaya woreda (synonymous with district), East Hararghe Zone, Oromia Region, Ethiopia. Detailed study area description has been published [18, 19]. Households in the selected kebeles maintain a variety of livestock (chickens, cattle, sheep, and goats) living in proximity with family members of the household. In some cases, the livestock co-habit with humans within the same room inside the house, often uncontained, to avoid issues with predators outdoors. Based on this information, human stool samples, livestock feces, and environmental samples were collected over time from each household to assess the prevalence and load of *Campylobacter*. Full details of the enrollment process and *Campylobacter* detection at the genus level have be previously described [18]. In short, a total of 1073 infant stool samples were collected monthly from 2 to 3 weeks after birth until 376 days of age. To minimize the external contamination, the first morning stools, minimum 10 g but sometimes as little as 1 g during the early months, were gathered in sterile plastic sheets within diapers that were sterilized with 12-h UV light either on the same or preceding day. These samples were then transferred using a sterile wooden spatula to 207-mL Whirl–Pak bags and transported on ice to the laboratory for processing. For infants, diarrhea presence on sampling days and the day prior to specimen collection, along with the 24-h loose stool count, were noted. Samples were stored in − 80 °C until further analysis.

### Sample selection for shotgun metagenomics

For the metagenomic analysis, the sampling frame was households (*n* = 106) that completed follow-up. Nine households that were initially included in the study did not complete follow-up: 4 households withdrew from the study, three infants died from cause unrelated to the study and two moved out of the study area [22]. We prioritized samples of good DNA quality, based on Nanodrop measurements (> 20 ng/µL, A 260/280 > 1.8 and A 260/230 < 1.5) and identified 50 households with at least one sample of each type. From these, we selected 40 households with balanced distribution across the 10 kebeles in the study area. We selected the latest available infant stool sample because our previous qPCR results [19] suggested the likelihood of detecting *Campylobacter* increased with age. We included one sample per household from each of the other types, preferring the sample closest in time to the infant sample if available (Table S1). In addition to the 280 study samples, two technical water control samples were included to monitor the integrity of the sample processing and metagenomic library preparation workflows.

### Extraction of genomic DNA and Campylobacter detection using qPCR

A QIAamp Power Fecal Pro DNA kit (QIAGEN, CA, USA) was used and briefly, 0.25 g of the sample, as recommended by the manufacturer, was used to extract genomic DNA from human stool samples and livestock feces. The quality and quantity of the DNA were analyzed using a UV5 Nanodrop spectrophotometer (Mettler Toledo, Columbus, OH). DNA with poor quality were cleaned using a Zymo genomic DNA clean and concentrator kit (Zymo Research, CA, USA) and stored at − 20 °C until further use. Aliquots of the DNA extracts were used for *Campylobacter* spp. detection at the genus level in the human stool samples and animal feces using TaqMan real-time PCR [14]. Detailed steps and results are described elsewhere [19].

### Library preparation and metagenomic sequencing

Library size and concentration were determined using the 4150 Tapestation system (Agilent, MA, USA). Limited-cycle PCR was subsequently employed to amplify the tagged DNA and introduce sequencing indexes. To facilitate a limit of detection assessment for each sample, we incorporated PhiX Control v3 (Illumina, Inc, CA, USA) into each sample prior to library preparation. The prepared libraries were loaded onto a reagent cartridge and subjected to clustering on the NextSeq 2000 System. Subsequently, a paired-end sequencing run with 2 × 150 bp reads was executed using the NextSeq 2000 platform. The base calls generated by the NextSeq 2000 System were then transformed into FASTQ files, facilitating downstream metagenomic analysis.

### FASTQ assessment for sample and process evaluation

We conducted an initial assessment of quality-related metrics, including cluster density, q-scores, and the percentage of passed reads as provided by the sequencer, using FastQC v.11.7 (Babraham Bioinformatics). Additionally, more comprehensive quality checks were conducted using the CZ-ID pipeline (mNGS Pipeline v7.1). This involved evaluating total reads, the percentage of high-quality reads in the total pool, and assessing sequence diversity through duplicate compression ratios. Total reads were utilized to estimate the read count per sample, while the “passed QC” metric represented the percentage of reads retained after applying quality filtering using fastp, which eliminated low-quality bases, short reads (< 35 bp), and reads with low complexity. To evaluate sequence diversity within the metagenomic samples, the duplicate compression ratio (DCR) was calculated for each sequencing run. DCR quantifies the prevalence of duplicate reads in a sample. A high DCR value, indicating numerous duplicate reads, suggests a less diverse library and account for potential technical artifacts affecting diversity assessments.

### Bioinformatic processing of the metagenomic data using the CZ-ID pipeline

The CZ-ID pipeline (Illumina mNGS Pipeline v7.1) was used for metagenomic analysis, incorporating a series of steps for thorough data cleanup and analysis refinement (Supplemental material S1). Host sequences were filtered, and ERCC sequences were excluded using STAR as the initial processing steps. Subsequently, we trimmed sequencing adapters and Illumina indexes through Trimmomatic. Additional filtering was then carried out using PriceSeq, enabling the retention of high-quality sequences using the Phred score values. Duplicate reads were identified and removed using czid-dedup. Following these steps, any residual host sequences were rigorously filtered out using Bowtie2. In cases where more than one million reads remained after this process (or two million for paired-end data), random subsampling was performed using unique or deduplicated reads. Finally, irrespective of host considerations, CZ ID filtered out human sequences using a combination of STAR, Bowtie2, and GSNAP, resulting in comprehensive data preparation and quality control pipeline. Sequences then subjected to metagenomic assembly to align and merge short sequencing reads obtained from a longer DNA sequence to reconstruct the original sequence. Initially, sequence alignment steps in CZ ID involved mapping reads to NCBI NT (nucleotide) and NR (protein) databases to obtain preliminary accession for each read and generating BLAST database containing all taxa identified as preliminary accessions. CZ ID implements SPAdes to assemble contigs. Each contig is composed of several sequencing reads and the pipeline maps quality-filtered reads back to the assembled contigs to determine which reads are associated with each contig using Bowtie2. This step involved aligning BLAST contigs against database of preliminary accessions generated in the previous steps. Finally, each read and the final accession (based on contig accession, or if the reads failed to assemble into contigs–based on the preliminary accession) mapped to taxa using NCBI accession to taxon database and generated compute statistics per each sample.

We employed downstream filtering steps to eliminate potentially erroneous taxonomic assignments. Drawing from our experience in formative research [15] and adhering to thresholds recommended by the developer [23] and established community best practices [15, 24–26]. Specifically, we used “NT L ≥ 50” to retain taxa with alignments of at least 50 base pairs in the nucleotide databases, “NT R ≥ 10” to preserve taxa with a minimum of 10 reads aligning to the nucleotide databases. Furthermore, to ensure data normalization and mitigate background noise, we incorporated a Z-score filter derived from a background model constructed using control samples used during DNA extraction (“Z-score > 1”; taxa abundance is higher in the sample than in the control; “Z-score = 100” taxa not found in the set of controls; “Z-score = − 100” taxa only present in the controls). The outcomes of our metagenomic pipelines were comprehensively evaluated for meeting all three filtering criteria mentioned above. This approach helped to enhance the reliability of our taxonomic assignments in the metagenomic dataset.

### Statistical analysis

Statistical analyses were performed using JMP PRO 16 software (SAS Institute, Cary, NC, USA). Metagenomic read counts across the seven different sample types were first assessed for normal distribution. To evaluate differences in mean sequencing depth among the sample types, a one-way analysis of variance (ANOVA) was performed. ANOVA was also employed to compare the mean values of alpha diversity metrics, specifically the Shannon and Simpson diversity indices. When significant differences were detected, Tukey’s Honest Significant Difference (HSD) test was used for post hoc pairwise comparisons. Additionally, the Shannon and Simpson diversity indices were compared using pairwise non-parametric comparisons (Wilcoxon test).

The Chi-square test was employed to compare the probability of detecting *Campylobacter* species across various kebeles. Additionally, pairwise comparisons were conducted to estimate the likelihood of co-infection for specific pairs of *Campylobacter* species detections. The abundance of each bacterium in the stool samples was estimated based on normalized read count, reads per million (rpm). Bi-variate analysis was utilized to examine the relationships between *Campylobacter* load from qPCR [19], with metagenomic abundance, rpm. A non-parametric Wilcoxon test was performed to identify rpm abundance differences for a given member of the microbial species. Hierarchal clustering (using Ward’s method), multivariate analysis (no-covariates were included) combined with Pearson pairwise correlation coefficient and principal component analysis, were employed to uncover patterns and associations within the microbiome dataset. For selected samples, with at least one *Campylobacter* contig, a pairwise distance matrix was generated by performing a kmer-based reference-free genomic distance estimation known as split k-mer analysis (SKA). For this analysis, contigs were compared based on matching short stretches of sequence and not to a designated reference sequence. Calculation of pairwise distances between sample clusters is based on defined SNP = 20 and identity (0.9) cutoffs [27, 28].

## Results

### Metagenomic sequencing depth and read distribution in the seven sample types

Examination of reads from stool samples (infants, mothers, and siblings), and fecal samples from livestock (cattle, goats, sheep, and chickens) demonstrated a mean sequencing depth of 1.06 × 10^8^ (95% CI, 1.02–1.09 × 10^8^), with highest sequencing depth of 1.5 × 10^8^ per sample. In Fig. S1A, the distribution of total sequencing depth achieved is illustrated, with a comparison of means across different sample types. Chicken feces exhibited the highest mean sequencing depth (1.2 × 10^8^ reads, 95% CI: 1.1 × 10^8^–1.2 × 10^8^) and the narrowest distribution, indicating consistent sequencing quality, while infant stool showed the lowest mean sequencing depth (9.8 × 10^7^ reads, 95% CI: 8.4 × 10^7^–1.0 × 10^8^) with greater variability. However, a one-way ANOVA test (*p* = 0.06) did not reveal significant differences in means. We also examined the complexity of sequencing reads in each sample as a measure of microbial biomass diversity by calculating the duplicate compression ratio (DCR), which represents the ratio of sequences before and after the removal of duplicated reads. Notably, all the samples exhibited a DCR ratio of < 2, underscoring the presence of diverse sequences and absence of biases from PCR amplification during the sequencing process (Fig. S1B).

### Detection of Campylobacter species in human stool and animal feces through shotgun metagenomics

**Fig. 1:**
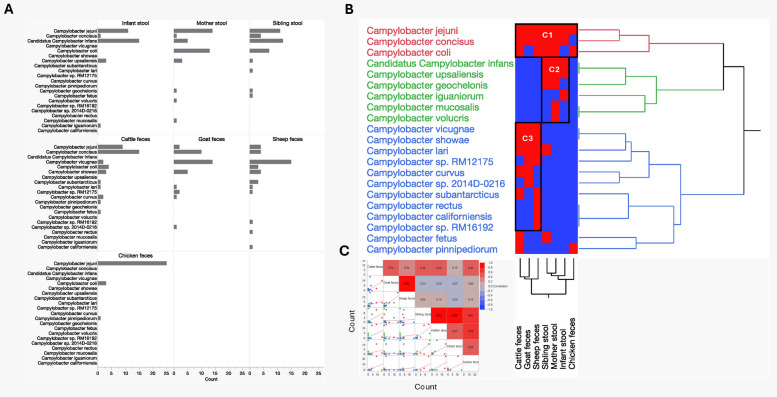
*Campylobacter* diversity in stool and fecal samples from seven sample types. **A** Bar plot showing diversity of *Campylobacter* species detected in human stool and livestock feces. Multiple *Campylobacter* species were detected in infants (*n* = 5), mothers (*n* = 8), and siblings (*n* = 8). Ruminant feces showed more *Campylobacter* species, with sheep (*n* = 11), cattle (*n* = 10), and goats (*n* = 8). Chicken samples showed the lowest diverse *Campylobacter* species (*n* = 3). **B** Two-way hierarchical clustering revealed distinct clusters in the detection of *Campylobacter* species across the seven sample types. Cluster C1, represents a broad-host range species, Cluster C2, human associated species and cluster C3, ruminant associated species (red cells represent detection and blue cells represent absence of detection). **C** Multivariate analysis of the detection frequencies of each *Campylobacter* species in different host types. The heat map shows red (strong correlation)/blue (no correlation)/green (negative correlation). Significant correlations were observed among *Campylobacter* species detected within human (infant, sibling and mother *r* > 0.64). Strong positive correlation between infant and chicken (*r* = 0.54), no to low correlation between infants with small ruminants (*r* = 0–0.15) and cattle (*r* = 0.19) respectively

Overall, with the exception of 17 samples (Human stool samples: infant (*n* = 1), sibling (*n* = 3), mother (*n* = 1) and livestock samples: cattle (*n* = 2), goat (*n* = 5), sheep (*n* = 2), and chicken (*n* = 3), excluded from analysis due to not meeting sequence quality thresholds, *Campylobacter* was detected in all remaining samples (*n* = 263/280). For thirteen of these samples, detection was restricted to the genus level, while in 250 samples, *Campylobacter* was identified at the species level. Figure 1A shows the frequency of *Campylobacter* species detected in stool (infants, mothers, and siblings) and fecal samples (cattle, goats, sheep, and chickens). In total, we detected 21 *Campylobacter* species across human and animal hosts. This includes the detection of multiple *Campylobacter* species in infants (*n* = 5), mothers (*n* = 8), and siblings (*n* = 8). In ruminant feces, the diversity of *Campylobacter* species varied, with sheep (*n* = 11), cattle (*n* = 10), and goats (*n* = 8) species. Chicken samples showed three *Campylobacter* species.

*C*. *jejuni* (*n* = 76, 30%) and *C*. *concisus* (exception for chicken feces, *n* = 49, 14%) were the most prevalent species detected, indicating their widespread presence in both human and animal hosts. Other highly prevalent species included *Candidatus* Campylobacter infans (*C*. *infans*), *C. vicugnae*,* C*. *coli*, and *C*. *showae*. Thermophilic *Campylobacter* species, including *C*. *lari* and *C*. *upsaliensis*, were also detected; however, their occurrence was infrequent, with a prevalence ranging between 1.6 and 2.8%. Other less frequently detected species include *C*. *iguaniorum* (infant), *C*. *mucosalis* (mother), *C*. *rectus* (sheep), *C*. sp. 2014D-0216 (goat), *C*. *californiensis* (sheep), *C*. *RM16192* (sheep), *C*. *volucris* (mother), and *C*. *pinnepideorioum* (chicken) (Fig. 1A).

### Campylobacter species in stool and fecal samples from different sources

Two-way hierarchical clustering found distinct patterns in the detection of *Campylobacter* species across the seven sample types (Fig. 1B). This analysis identified three major clusters among the 21 *Campylobacter* species. The first cluster, labeled C1, represents a mixed-host group, including species such as *C*. *jejuni*, *C*. *concisus*, and* C*.* coli*, which were detected in both human and livestock samples. The presence of these species in different sample types indicate niche overlap for these *Campylobacter* species. The second group is human associated cluster, labeled as C2 and includes *Campylobacter* species exclusively found in human samples. It included *C*. *infans* and *C*. *upsaliensis*, which are present in infant, mother and sibling stools. Other species, such as *C*. *geochelonis*, was detected in sibling and mother stool, while *C*. *mucosalis* and *C*. *volucris* were found in mother samples, and *C*. *iguaniorum* was identified only in infant samples. The third group is a ruminant associated cluster labeled as C3, containing 12 *Campylobacter* species, among them the recently described species *C*. *vicugnae*, and *C*. *californiensis* [29, 30], as well as long established species such as *C*. *showae* and *C*. *lari* (also detected in sibling stool) were detected. Unlabeled as clusters, *C*. *fetus* and *C*. *pinnipediorum* were each detected in only single instances per sample type. *C*. *fetus* was identified in a sample from a sibling and in cattle. Meanwhile, *C*. *pinnipediorum* was exclusively detected in chicken and cattle feces.

Notably, as shown in Fig. 1C, these clusters are driven by a strong positive correlation in *Campylobacter* species detection among ruminant samples (*r* > 0.34) and within human samples (infant: sibling, *r* = 0.88, infant: mother, *r* = 0.57), indicating a high degree of species overlap within these groups. Additionally, *Campylobacter* species from chicken samples showed a strong correlation with human samples (chicken: infant *r* = 0.54, chicken: sibling, *r* = 0.61, chicken: mother, *r* = 0.76) but only a moderate correlation with cattle samples (*r* = 0.46). There is no significant correlation observed between *Campylobacter* species from chickens and small ruminant samples (*r* = 0 and 0.15, goat and sheep, respectively), suggesting distinct *Campylobacter* profiles in these groups.

### Multiple Campylobacter species co-occur in stool and fecal samples

**Fig. 2:**
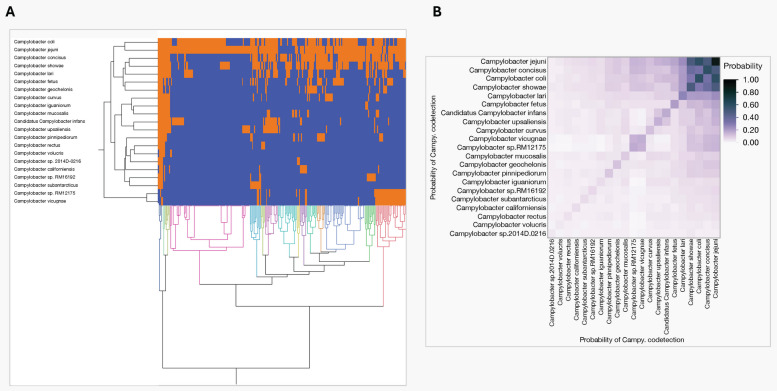
Hierarchical clustering heatmap displaying co-occurrence relationships among various *Campylobacter* species across stool and fecal samples from seven sample sources. **A** Columns represent individual samples, and rows correspond to different *Campylobacter* species. *C*. *jejuni*, *C*. *concisus*, and *C*. *coli*, showing notable co-occurrence patterns across multiple stool and fecal samples. In contrast, species like *C*. *vicunae* and *C*. sp. *RM12175* demonstrate distinct co-occurrence restricted to small ruminant samples. **B** Pairwise comparisons revealed a co-occurrence likelihood exceeding 0.5 in certain species pairs (*C*. *jejuni *and* C*. *coli*), (*C*.* jejuni *and* C*. *concisus*), and (*C*. *jejuni* and* C*. *showae*)

A key advantage of the metagenomic approach is its ability to investigate co-infections across all samples and seven sample types, enabling profiling of both dominant and less abundant *Campylobacter* species within sample. Our findings show a broader range of *Campylobacter* species other than the 21 most dominant ones identified across the seven sample types (Table S2). Complete list of *Campylobacter* species detected in individual samples are shown in Table S3. For the purpose of co-infection analysis, we limited our focus to the 21 most dominant *Campylobacter* species described in the previous section. Interestingly, the presence of distinct clusters along the y-axis indicate that *Campylobacter* species are commonly found together across multiple samples (Fig. 2A). Species such as *C*. *jejuni*, *C*. *concisus*, and *C*. *coli* that were widely represented showed notable co-occurrence patterns across multiple stool and fecal samples. In contrast, species like *C*. *vicugnae* and *C*. sp. *RM12175* demonstrate distinct co-occurrence patterns, particular to small ruminant samples. Using probability co-occurrence matrix, we identified specific pairs of *Campylobacter* species in various stool and fecal samples from different sources with higher likelihood of co-occurrence (*C*. *jejuni and C*. *coli*), (*C*. *jejuni *and* C*. *concisus*) and (*C*. *jejuni *and* C*. *showae*) with a co-occurrence probability of (Pr > 0.5) (Fig. 2B).

### Abundance of key Campylobacter species in stool and fecal samples and correlation with qPCR detection

**Fig. 3:**
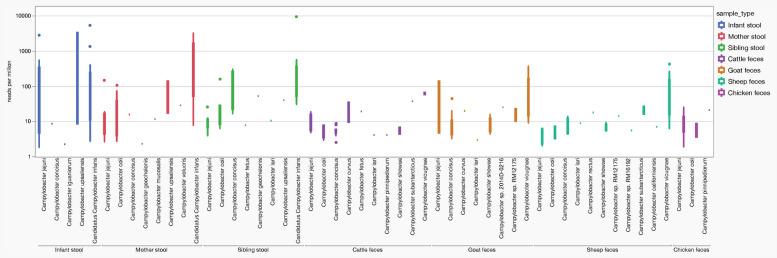
*Campylobacter* abundance across seven sample types measured as reads per million (rpm). Samples with rpm values exceeding 1000 were exclusively from human sources, including infant stool. Among human samples, *Candidatus* Campylobacter infans exhibited significantly higher rpm compared to *C*. *jejuni*, *C*. *concisus*, *C*. *coli*, and *C*. *lari* (*p* < 0.05, Wilcoxon test). In ruminant samples, although diverse Campylobacter species were detected, overall reads per million was significantly lower than in human samples, with the exception of goat samples (*p* < 0.05, Wilcoxon test)

*Campylobacter* abundance across seven sample types was investigated using a standardized nucleotide count, reads per million (rpm) (Fig. 3). Notably, samples with NT.rpm exceeding 1000 were exclusively derived from humans, including the infant stools Fig. 3. Among these human samples, *Candidatus* Campylobacter infans exhibited significantly higher rpm values compared to *C*. *jejuni*, *C*. *concisus*, *C*. *coli*, and* C*. *lari* (*p* < 0.05, non-parametric multiple comparison (Wilcoxon test).

In ruminants, despite the presence of diverse *Campylobacter* species, NT abundance, as measured by rpm, was notably lower than in human stools and significant difference was observed in the median rpm between infants and the livestock samples with the exception of goat samples (*p* < 0.05, non-parametric multiple comparison (Wilcoxon test).

Sliding window analysis further demonstrated concordance between metagenomic read abundance (rpm) and the Ct values of genus level qPCR assay used for *Campylobacter* detection, which was previously reported by [19] (Fig. S2).

### Genetic relationships of selected Campylobacter species across diverse sample types

**Fig. 4:**
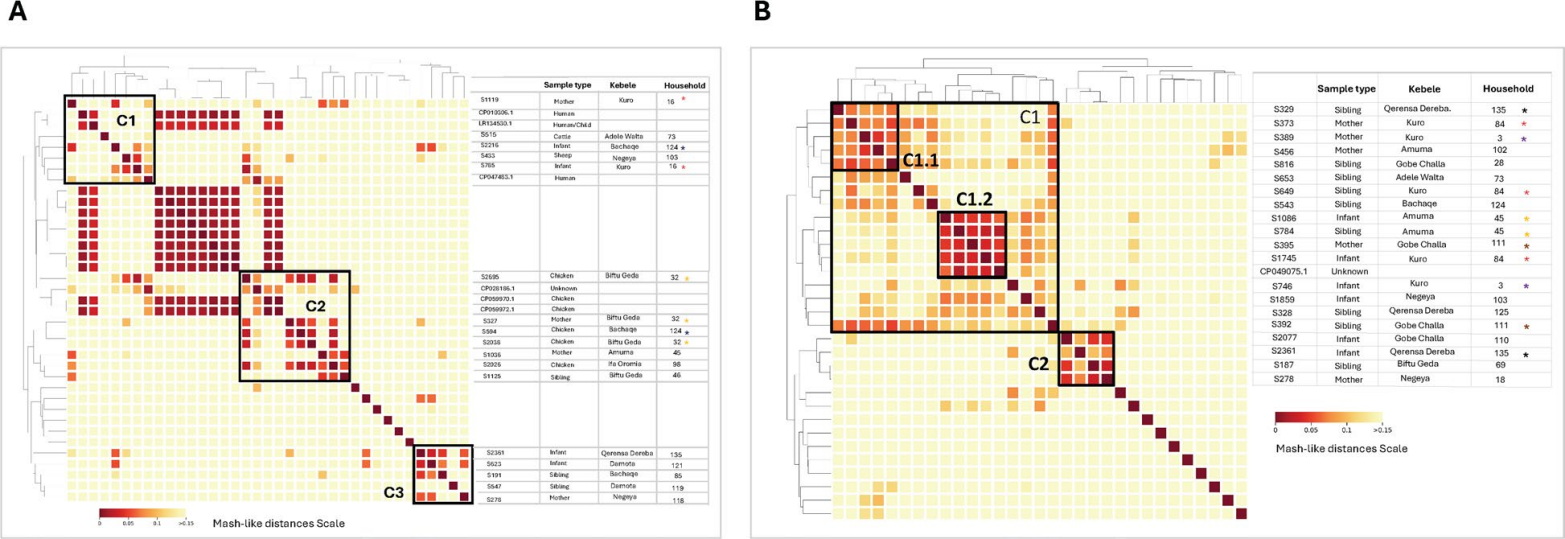
Pairwise Mash-like distances for *Campylobacter* species in a matrix format. **A** *Campylobacter jejuni*; **B** *Candidatus* Campylobacter infans. Each sample in the dataset was compared to itself and all other samples. The colors in the matrix represent the range of Mash-like distances (refer to the scale). The diagonal line compares each sample to itself, resulting in dark red squares, indicating zero differences between the sequences. Black boxes are used to show clustering of samples (labeled as C1-3), unlabeled cluster in (**A**) between C1 and C2, are reference samples from database used in the clustering. In (**B**), C1.1 and C1.2 represent subclusters. On the right side of (**A** and **B**), sample metadata, including host, Kebele, and household, utilized to support the genetic similarities observed in the metagenomic dataset. Asterisks (*) denote samples collected from the same household

We investigated genetic similarities of selected *Campylobacter* species, specifically focusing on most widely detected species across the seven sample types: *C*. *jejuni*, *C. concisus*, *C*. *infans*, and *C*. sp.* vicugnae*. For *C*. *jejuni*, three major clusters (C1-3) were identified, shedding light on genetic similarities of circulating species (Fig. 4). Cluster C1 includes *C*. *jejuni* exhibiting phylogenetic relationships with known reference isolates, specifically *Campylobacter jejuni subsp*. *jejuni* strain:00–1597 (Accession ID_CP010306) and *Campylobacter jejuni subsp*. *doylei* NCTC 11924 [LR134530]. These isolates are associated with human enteritis, with CP010306 isolated from a human in Canada [31] and sequence submitted from a child with diarrhea in the UK. Additional samples in C1 cluster include samples primarily originating from human sources and two of the samples (1119, mother and 785, infant) were from the same household 16. C1 also includes samples from cattle and sheep sources, suggesting potential cross transmission of *C*. *jejuni* across human and ruminant hosts.

Similarly, in C2, the *C*. *jejuni* cluster is predominantly constituted from chicken sources. The three reference strains identified in this cluster include *C*. *jejuni* strain CFSAN054107 (accession: CP028185.1) from unknown source and *C*. *jejuni* strain C57 and strain C220 (with accession CP059970.1 and CP059972.1 respectively), derived from the cloacal swab of a chicken host. Overall, this cluster exhibits some host structuring, with samples from chicken showing genetic relatedness as measured in the relative distance (Mash-like distances < 0.1) with each other. In addition, the C2 cluster also showed a spatial signature with four of these sample being from the same kebele (Biftu Geda) and three of the four also being from the same household. The three samples that shared household were from mother and chicken source, indicating potential cross transmission of *C*. *jejuni* between hosts. In contrast, C3 showed host structuring with all *C*. *jejuni* in the group coming from human stool including two infant sources.

For *Candidatus Campylobacter infans*, as shown in Fig. 4B, the data reveals a cluster encompassing stool samples from infants, mothers, and siblings. This cluster is divided into two main groups, C1 and C2, with further subdivisions into subclusters C1.1 and C1.2, where household level concordance was observed for some of the samples within these clusters. In Cluster C1, samples from Households 3, 45, 84, and 111 demonstrate genetic clustering of *Candidatus* Campylobacter infans. Specifically, in Household 3, the samples represent an infant and a mother; in Household 45 (located in Amuna Kebele, within C1.2), infant and sibling stool samples show genetic similarity. Additionally, Household 84 in Kuro Kebele contains clustered stool samples from a mother and a sibling, and Household 111 includes samples from a mother and a sibling as well. These instances of genetic and spatial clustering suggest potential household-level cross transmission of *Candidatus* Campylobacter infans. Furthermore, in Household 135, as illustrated in Fig. 4B, stool samples from an infant and a sibling appear genetically divergent despite the shared household environment. This finding suggests the presence of wider genetic diversity of *Candidatus* Campylobacter infans*,* with strains circulating in the same spatial setting.

For *C*. *concisus* two clusters, C1 and C2, were identified (Fig. S3A), with notable host structuring within each. Cluster C1 comprised *C*. *concisus* isolates predominantly from small ruminants (sheep and goats), whereas cluster C2 grouping, was primarily associated with sibling and maternal stool samples. In contrast, *C*. *vicugnae* was identified only in goat and sheep sources, and genetic variation was observed within small ruminant hosts (Fig. S3B).

### Comparative analysis of stool microbiome diversity and shared bacterial communities across seven sample types

Following the application of metagenomic filters, our analysis detected more than 3844 genera. Overall, the microbial composition showed a consistent bacterial dominance in the microbiota composition, with variations in other kingdoms depending on sample type. Bacterial communities constituted over 85% of sequences across all groups, with sheep and goat feces having the highest bacterial detection (92.49% and 92.91%, respectively). Eukaryote presence varied, ranging from 4.56% in goat feces to 10.38% in chicken feces. Archaeal communities were most abundant in cattle feces (2.97%), while infant stool exhibited the lowest archaeal presence (0.26%). Viral representation (mostly phages), though minimal in most samples, was notably higher in infant stool (5.52%) and sibling stool (3.06%) compared to other groups, where viruses ranged from 0.29 to 2.35%.

**Fig. 5:**
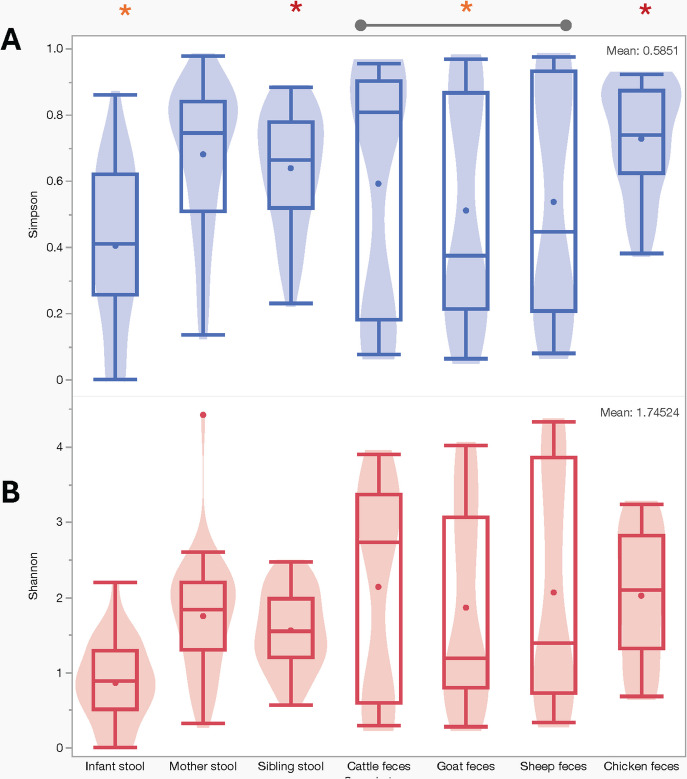
Alpha diversity of microbial communities across seven sample types, measured by the Simpson (**A**) and Shannon (**B**) diversity indices. In **A** and **B**, (*) used to show significant association one sample type with rest of the sample type; (*) used show significant association between two sample types. Ruminant (cattle, goats, and sheep) samples (*), display higher microbial diversity and evenness, indicated by broader distributions and higher mean in both Simpson and Shannon indices (ANOVA, Shannon and Simpson, *p* < 0.001). Tukey’s HSD indicated that infant stool (*) samples exhibited significantly lower microbial diversity and richness compared to all other sample types (Tukey HSD for all pairs, *p* < 0.05). Additionally, using pairwise non-parametric comparisons, a statistically significant difference was found between sibling stool and chicken fecal samples (*) (Wilcoxon test: *p* = 0.015)

Comparisons of means for alpha diversity indices (Shannon and Simpson diversity) revealed significant differences in microbial diversity and evenness across the seven sample types (ANOVA, Shannon and Simpson, *p* < 0.001; Fig. 5). Further pairwise comparisons using Tukey’s HSD indicated that infant stool samples exhibited significantly lower microbial diversity and richness compared to all other sample types (Tukey HSD for all pairs, *p* < 0.05). Recognizing that Tukey’s HSD may overlook more nuanced differences in diversity among sample types, we additionally conducted pairwise non-parametric comparisons using the Wilcoxon method. This analysis revealed a statistically significant difference between sibling stool and chicken fecal samples (Wilcoxon test: *p* = 0.015), and all pairwise comparisons involving infant samples also yielded significant differences (*p* < 0.05).

**Fig. 6:**
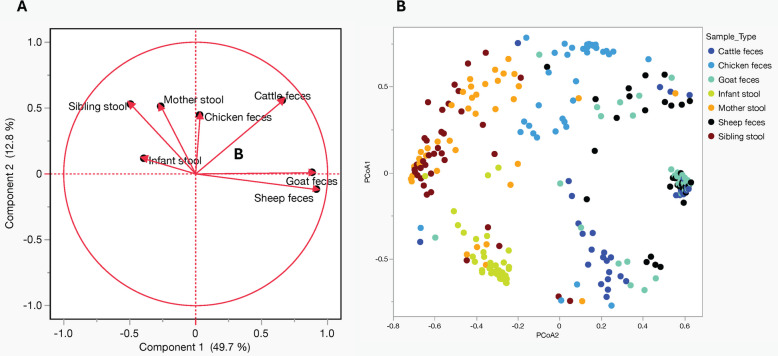
Principal component analysis (PCA) based on microbial community composition: The plots show clustering of microbial communities from different sample types based on beta diversity. **A** Microbiota diversity was explored through principal component analysis (PCA), revealing two major components explaining 62.5% of the microbial diversity, attributed to sample types. PCA highlighted distinct microbiota clustering within ruminant samples, notably influenced by small ruminant samples in component 1. Similarly, microbiota clustering among human samples was explained by component 2, with chicken microbiota displaying similarities to humans and contributing mainly to component 2. **B** Each point represents a sample, color-coded by sample type as indicated in the legend. The axes represent the principal coordinates (PCoA1 and PCoA2), which explain the variation in microbial community structure between samples. Samples that are closer together share more similar microbial compositions, while those farther apart are more dissimilar. The distinct clustering of colors reflects differences in microbial communities across sample types

For b-diversity, we used, principal component analysis (PCA) that differentiated the microbial communities into two main components explaining 62.5% of the total diversity (*p* < 0.001) being as a result of sample types (Fig. 6A). Component 1 explained approximately 50% of the diversity in the total samples. This component consisted of 92% from the microbial community in sheep, 88% in goats, and 66% in cattle, with a minority of 0.03% in chicken. This demonstrates the substantial contribution of ruminant samples. In contrast, component 2, which explained about 13% of variability within the samples, was loaded from human stool (53% in siblings, 51% in mothers, and 11% in infants) and 44% from chicken feces. Furthermore, Fig. 6B explains these differences by showing the relationship of individual sample based on variation in microbial community structure across the seven sample types as denoted by clustering of similar colors.

**Fig. 7:**
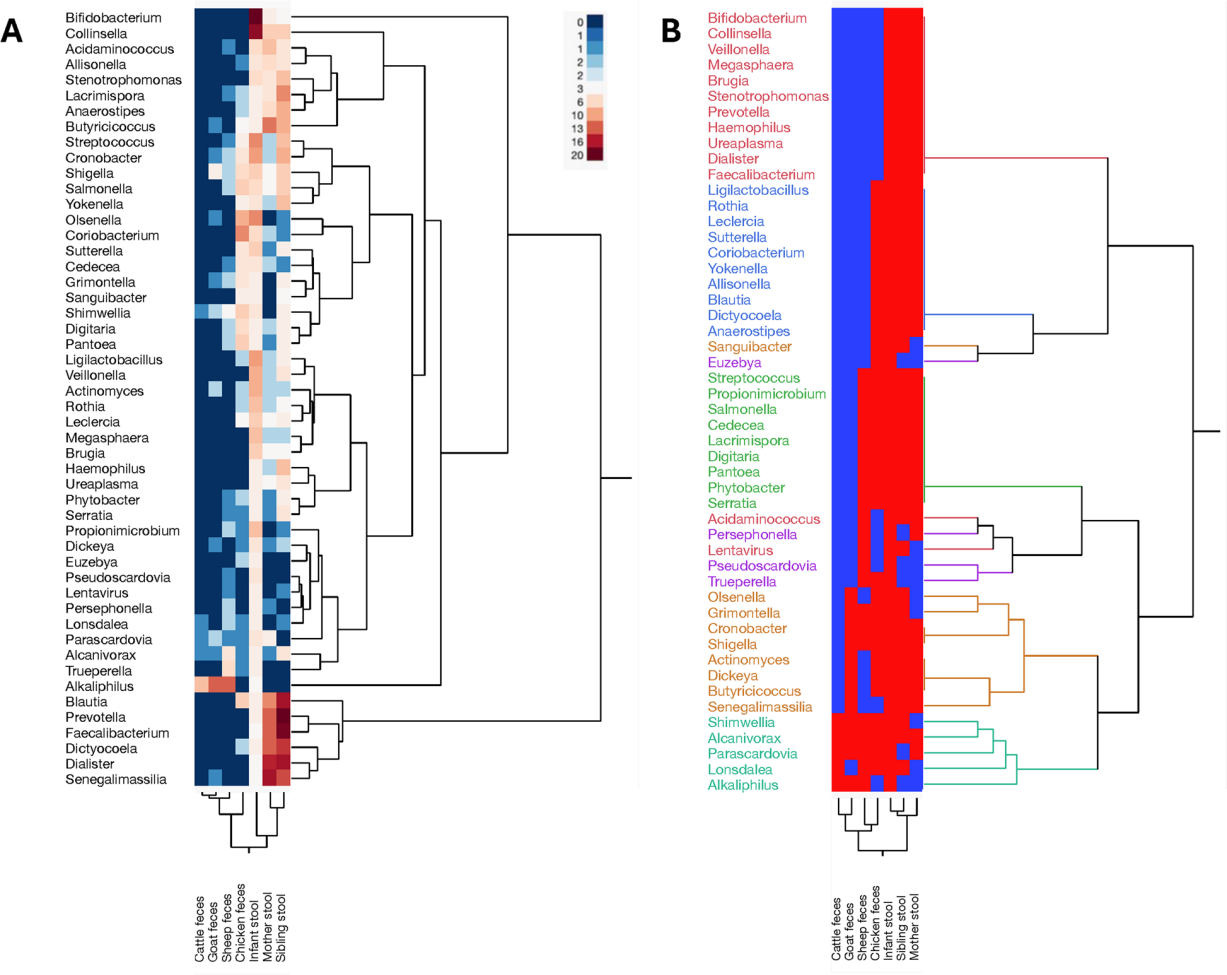
Top 50 abundant organisms in infant stool. **A** Heat map with color gradient represents relative abundance of each microbial genus, with darker red indicating higher abundance and darker blue representing lower abundance or absence. The dendrograms on the right and bottom illustrate hierarchical clustering based on the similarity of microbial composition across taxa and sample types, respectively. **B** Heat map displaying the hierarchical clustering based on presence (red) or absence (blue) of organisms across different sample types. The dendrograms on right and bottom represent the similarity between microbial taxa and, sample type respectively, with more closely related clusters grouped together

Notably, this analysis highlighted similarities between the microbial profiles of infant stool and chicken samples compared to other livestock samples, with maternal stool showing the closest relationship to the infant (Fig. 6A). Cluster analysis from the Bray Curtis dissimilarity metrics indicated that while infant stools shared a core microbiome with maternal and sibling samples, there was also a more pronounced compositional divergence. The infant microbiome is distinctly characterized by a high presence of *Bifidobacterium, Collinsella, Streptococcus*, and *Olsenella* species (Fig. 7A). These four genera are considerably less prevalent in the stools of mothers and siblings, and are absent in livestock samples, except for a single detection of *Streptococcus* spp.in sheep feces which underscores the differences in microbial composition between infants and mothers and siblings.

Analysis of the top 50 most abundant microbial taxa in infant stool revealed a distinct cluster uniquely present in human stool samples and absent in all livestock samples. This cluster includes the genera *Bifidobacterium*, *Collinsella*, *Veillonella*, *Megasphaera*, *Bruquia*, *Stenotrophomonas*, *Prevotella*, *Haemophilus*, *Ureaplasma*, *Dialister*, and *Faecalibacterium*. Chicken feces exhibited microbial similarities with human stool samples (infant, mother, and sibling), sharing genera such as *Ligilactobacillus*, *Rothia*, *Leclercia*, *Sutterella*, *Coriobacterium*, *Yokenella*, *Allisonella*, *Blautia*, *Dictyocoela*, and *Anaerostipes*. Additionally, *Euzebya* was shared exclusively with infant stool, while *Sanguibacter* was shared with infant and sibling stools. In contrast, genera such as *Shumwellia*, *Alcanivorax*,* Lonsdalea*, and *Alkaliphilus* appeared variably across most sample types (Fig. 7B).

### Sub-group analysis of enteric microbiome based on findings from the MAL-ED and GEMS studies

**Fig. 8:**
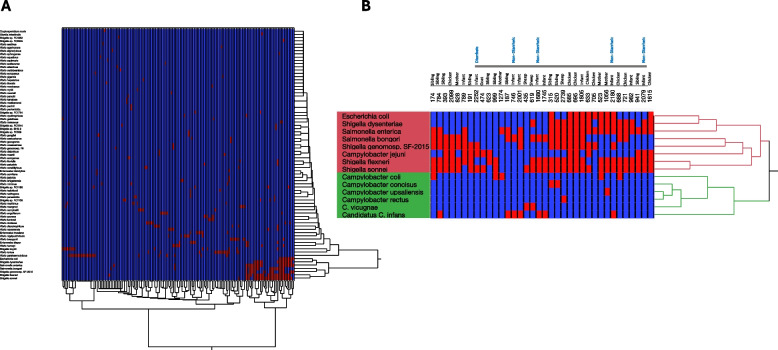
Hierarchical clustering heat maps showing the presence of selected enteric pathogens across samples. This figure represents the presence and absence of key enteric pathogens in various samples, providing insights into co-occurrence patterns of species. The selection of pathogens included in the analysis was informed by the results of MAL-ED, GEMS studies, and WHO FERG estimates, focusing on pathogens known to impact infant health in developing countries. **A** Heat map showing the presence of enteric species across different samples (columns represents individual samples, red cells indicating the presence of a pathogen and dark blue cells representing its absence. **B** Hierarchical clustering analysis identifies groups of organisms that tend to co-occur mostly across human and chicken samples. Notably, *Escherichia coli*, *Salmonella enterica*,* Salmonella bongori*, three species of* Shigella* (*S*. *flexneri*, *S*. *sonnei*, and *Shigella* sp. *genomosp*. *SF*-*2015*), and *Campylobacter jejuni* form a cluster of species with higher co-occurrence patterns compared to other pathogens. Additionally, one infant sample with a history of diarrhea was found to harbor three species of *Shigella* as well as *Campylobacter jejuni*, whereas four other infant samples clustered in this group showed no history of diarrheal illness

This sub-group analysis investigated the presence and co-occurrence of selected enteric pathogens across seven sample types, informed by pathogen selection at the genus level based on findings from the MAL-ED and GEMS studies and WHO FERG estimates, which highlight pathogens of concern for infant health in developing regions [1, 6]. The analysis focused on common enteric pathogens, *Shigella*, *Vibrio*, *Salmonella*, *Entamoeba*, *Cryptosporidium*, and *Giardia*. Hierarchical clustering revealed distinct co-occurrence patterns among these pathogens, including *C*. *jejuni*. These bacterial co-occurrence patterns involving eight species, *Salmonella enterica*, *Salmonella bongori*, *Shigella flexneri*, *Shigella sonnei*, *Shigella* sp. *genomosp*. *SF-2015*, and *C. jejuni* showed a high tendency to co-occur across various sample types, particularly in human stool and chicken feces (Fig. 8A). Based on reported cases of infant diarrhea (as reported by the mother) within the clustered samples, co-detection of three *Shigella* species alongside *C*. *jejuni* in one diarrheal sample was observed. In contrast, four additional infant samples within the same cluster had no reported diarrheal history (Fig. 8B).

**Fig. 9:**
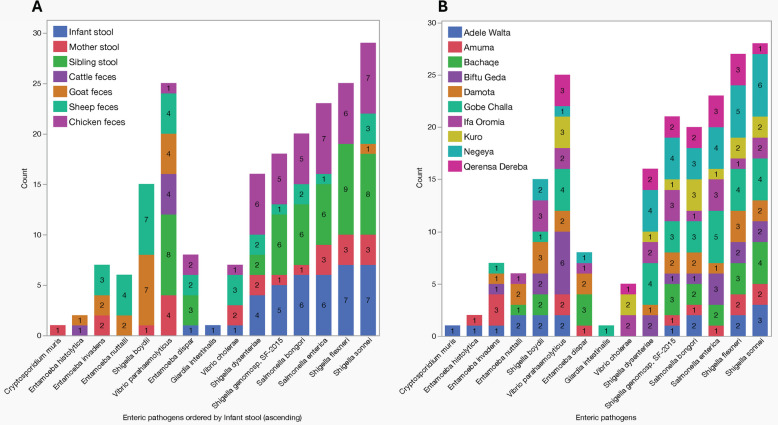
Distribution of selected enteric pathogens identified from various sample types, **A** human stools (infants, mothers, and siblings), animal fecal samples (cattle, goats, sheep, and chickens),** B** distribution of enteric pathogens across the study Kebeles. The numbers in each bar represent the number of positive detections of the pathogen in the corresponding sample type (**A**) and Kebele in (**B**)

In total, *Salmonella* spp. (*S*. *enterica *and *S*. *bongori*) was detected in 30 samples, with a detection rate of 11.5%. Both species co-occurred in 13 samples, and their presence was most notable in chicken feces, as well as in stool samples from infants and siblings. *Shigella* spp. were identified in 20.7% of samples (*n* = 54), with the four major species: *S*.* dysenteriae*, *S*. *flexneri*, *S*. *sonnei*, and* S*. *boydii* comprising 71% of detections. *S*. *flexneri* was the most prevalent species, with *Shigella* spp. detected across all sample types except for cattle, and the highest burden observed in infant stool (*n* = 31), sibling stool (*n* = 30), and chicken feces (*n* = 28) (Fig. 9A and B).

*Vibrio* spp. was detected in 117 samples across all sample types, representing 56 species, with *Vibrio cholerae* found in a limited number of samples (*n* = 7), including sheep feces (*n* = 3), and stool samples from mothers (*n* = 2) and infants (*n* = 2). *Vibrio parahaemolyticus* (*n* = 25) was frequently detected across a broader range of sample types, though there was no co-detection of *V*. *cholerae* and *V*. *parahaemolyticus* (Fig. 9A and B).

Among parasitic agents, *Entamoeba* spp. was detected in 20 samples, with species including *E*. *histolytica*, *E*. *dispar*, *E*. *invadens*, and *E*. *nuttalli*. *Cryptosporidium muris* was found in a mother stool, while *Giardia intestinalis* was detected in an infant stool sample (Fig. 9A and B). Viral detection was minimal overall, with *Human mastadenovirus* found in both infant and sibling stool samples. Phage-related viral detections were common, with notable exceptions were *Parapoxvirus*, Orf virus, in ruminant samples and *Gallid herpesvirus 1* in chicken samples. The Orf virus had the highest overall detection (*n* = 19), particularly in goat feces (*n* = 12).

## Discussion

*Campylobacter* infection is a significant cause of diarrheal illness among children under five in LMIC including Ethiopia [13, 32–34]. Systematic reviews and meta-analysis indicate that around 10% of children in this age group are affected by *Campylobacter*, with prevalence in animals reaching 14.6% compared to 9% in humans, suggesting its zoonotic nature [35–37]. Prior research primarily focused on thermophilic *Campylobacter* species like *C*. *jejuni*, *C*. *coli*, *C*. *lari*, and* C*. *upsaliensis* found in humans and domestic animals, while the prevalence of non-thermophilic species, such as *C*. *hyointestinalis*, *Candidatus C*. *infans*, *C*. *fetus*, *C*. *showae*, and* C*. *concisus*, remains largely unexamined.

This study leveraged samples from a cohort of 106 infants in rural eastern Ethiopia, representing households across 10 of Haramaya’s 36 rural kebeles, for shotgun metagenomic sequencing. A recent report from our group indicated detection by qPCR of *Campylobacter* at the genus level in 64% of infant stool samples, with prevalence increasing with age as reported previously [19]. By analyzing stool samples from infants, their associated environment (mothers, siblings, and livestock feces (from cattle, goats, sheep, and chickens), we captured a detailed view of *Campylobacter* infection dynamics and other enteric pathogens across multiple hosts, shedding light on potential pathways of early life colonization.

Metagenomic sequencing identified 21 major *Campylobacter* species across different sample types. Among these, five species, *C*. *jejuni*, *C*. *concisus*, *C*. *coli*, *C*. *infans*, and *C*. *upsaliensis* were found to colonize infants. The result highlighted early exposure of infants to diverse *Campylobacter* strains from both human (mother and sibling) and livestock sources, highlighting the effectiveness of metagenomic approaches in characterizing *Campylobacter* diversity and colonization patterns often missed by traditional methods [38, 39]. Notably, the detection of species like *C*. *concisus* and* C*. *infans*, especially in infants, is significant; *C*. *infans* is a relatively new proposed species, while *C. concisus* has been linked with chronic gastrointestinal conditions such as inflammatory bowel disease and has been observed in cases of gastroenteritis particularly in young children and immunocompromised patients [40–42]. Likewise, *C*. *infans* was identified as the second most dominant species in breastfed infants in a global enteric multicenter study and has been associated with diarrhea in humans and non-human primates [43]. In this study, *C*. *infans* was widely detected in human samples, including infants, and exhibited significantly higher read counts (rpm) values compared to *C*. *jejuni*, *C*. *concisus*, *C*. *coli*, and *C*. *lari*. A re-analysis of the GEMS dataset by [44] showed young children with diarrhea tended to have higher *Campylobacter* quantification in stool than asymptomatic controls, indicating a quantity-dependent effect [44]. Additionally, findings from our group in the same birth cohort showed that *Campylobacter* detection (qPCR) increased with age, and infants with higher average *Campylobacter* loads experienced diarrhea more frequently [19, 22].

Additionally, *C*. *upsaliensis* is relatively rare in infants, with its isolation often associated with dogs and cats [45, 46]. However, these findings originate from developed countries, where both healthy and sick dogs and cats have been identified as sources of *C*. *upsaliensis* infection in humans [47]. In the African contexts, research on *C*. *upsaliensis* is limited. A study in a rural South African setting did report the presence of *C*. *upsaliensis* in dogs, indicating that pets could similarly act as reservoirs in African regions [48]. However, overall, data supporting *C*. *upsaliensis* reservoir hosts in rural Africa setting remains sparse.

The study also found host specific and mixed host infection patterns among *Campylobacter* species. The detection of *C*. *jejuni* across all sample types underscores its ecological versatility and adaptive potential, aligning with previous findings on the zoonotic nature of this species [49, 50]. *C*. *jejuni* is well recognized for its host diversity and environmental resilience, which contribute to its status as one of the most prevalent causes of bacterial gastroenteritis worldwide [38, 51]. The mixed host clustering of *C*. *coli *and* C*. *concisus* also highlights their potential for cross-host transmission. Although *C*. *coli* is primarily associated with livestock, particularly pigs and poultry, it has been increasingly detected in humans and other animal hosts, indicating its adaptability and potential zoonotic threat [52, 53]. In this study, *C*. *coli* was detected in both infant stool and goat feces, reflecting its potential for mixed-host infection where direct or indirect contact between humans and livestock may have facilitated transmission. On the other hand, *C*. *concisus* was detected in all sample types except chicken feces, pointing to its relatively broad host range with a preference for mammalian hosts.

A major finding in this study is also the co-infection patterns among *Campylobacter* species which found significant associations, particularly for *C*. *jejuni*, demonstrating a high probability (Pr > 0.5) of co-occurrence with *C*. *coli*, *C*. *concisus*, and *C*. *showae*. These observations indicate the potential for interactions within the host environment, where multiple *Campylobacter* species may simultaneously colonize, potentially leading to complex infection dynamics influencing disease conditions in infants. These co-infections are likely due to synergistic effects or shared virulence mechanisms that facilitate persistence and survival within host microbiomes [54–56]. High co-occurrence probability of these pairs highlights the need for further investigation into the implications of such mixed *Campylobacter* infections in infant health.

In addition to the *Campylobacter* species, our study showed that *C*. *jejuni* exhibited strong clustering with other enteric pathogens, including *Salmonella enterica*, *Salmonella bongori*, and multiple *Shigella* species (*S*. *flexneri*, *S*. *sonnei*, and* Shigella *sp.* genomosp. SF*-*2015*). This clustering was particularly pronounced in human stool and chicken feces samples, suggesting shared environmental or dietary transmission routes. The frequent co-detection of *C*. *jejuni* with these pathogens aligns with other studies that have observed overlapping transmission pathways and reservoirs, particularly in resource-limited settings where sanitation and food safety practices are less stringent [57, 58]. Although the identification of *Shigella* spp. in animal hosts is noteworthy, the close genomic similarity between *Shigella* and *E*. *coli* suggests that these detections may reflect the presence of *E*. *coli* strains. Even though the metagenomic investigation in this study was centered on *Campylobacter* and key enteric pathogens, it was also complemented by a global microbiome analysis that identified two distinct microbiota clusters. The first cluster (principal component) associated with ruminants and another consisting of human and chicken samples. Using PCA, we demonstrated that the chicken microbiome shares organisms including enteric pathogens with the infant microbiome while the ruminant microbiome is more distinct. Using multivariate analysis, we found that *Campylobacter* species from chicken samples show a strong correlation with human stool samples (*r* > 0.54) but only a moderate correlation with cattle (*r* = 0.46). Further analysis of *C*. *jejuni* sub-clusters suggested that human and chicken samples often grouped closely, facilitating cross-host transmission between chickens and infants. In conclusion, the study highlights cross-host transmission potential of *Campylobacter* species with significant correlation among the *Campylobacter* species detected in human stools, suggesting that infants may acquire *Campylobacter* through exposure to colonized siblings or mothers. Additionally, the strong association with chickens points to their likely role as a direct source of human infection, while cattle may serve as a contributing reservoir to the broader environmental pool of *Campylobacter* in shared, multi-host settings.

### Limitations

This study has a few limitations worth noting. Since we focused on ten rural kebeles in eastern Ethiopia, the findings may not fully capture the range of environments and community conditions across the region. Our use of DNA-based metagenomics means RNA viruses were not included, so some viral players may have been missed. Even with deep sequencing (100 million per sample), low-abundance organisms posed a challenge, we could not always assemble genomes or trace how microbes might move between hosts. While we took steps to minimize contamination and ensure consistent sample processing, some variation in DNA extraction across different sample types could have influenced the results.

## Supplementary Information

Additional file 1: Fig. S1. Metagenomic sequencing depth and read distribution in seven sample types. A) Density plots showing the distribution of total sequence reads across different sample types. Each curve represents the distribution of sequence read counts for each sample type, with mean values, standard deviations, and 95% confidence intervals (CI). B) The duplicate compression ratio, denoting the ratio of sequences before and after duplicate sequence removal. Values less than 2 conforms the metagenomic sequencing criteria, affirming the absence of sequencing biases.

Additional file 2: Fig. S2. Correlation between *Campylobacter* metagenomic relative abundance and their detection through qPCR. Sliding window analysis depicts the correlation between metagenomic read per million with the genus-level qPCR Ct values. The analysis revealed a Pearson correlation coefficient of -53% (95% CI: -0.68 to -0.31), demonstrating agreement between the metagenomic relative abundance and genuslevel qPCR Ct values.

Additional file 3: Fig. S3. Pairwise Mash-like distances for A) *Campylobacter Concisus*; B) *Campylobacter vicugnae*. Each sample in the dataset was compared to itself and all other samples. The colors in the matrix represent the range of Mash-like distances (refer to the scale). The diagonal line compares each sample to itself, resulting in dark red squares, indicating zero differences between the sequences. Black boxes are used to show clustering of samples (C1 and C2 in A), and on the right side, sample metadata, including samples and host species are utilized to support the genetic similarities observed in the metagenomic dataset.

Additional file 4: Table S1. Details of sample selection for the metagenomic studies.

Additional file 5: Table S2. Twenty-one most dominant *Campylobacter* species identified across the seven sample types.

Additional file 6: Table S3. Complete list of *Campylobacter* species detected in this study across the sample types; shaded species are minor species detected in addition to the 21 major *Campylobacter* species.

Additional file 7: S1. The CZ-ID pipeline (Illumina mNGS Pipeline v7.1) was used formetagenomic analysis.

Additional file 8. S1. Biosample IDs for metagenomes.

## Data Availability

The metagenomics data is submitted to NCBI under Bioproject number PRJNA843963.
