# Supplementary figures and images for: Host clustering of Campylobacter species and enteric pathogens in a longitudinal cohort of infants, family members and livestock in rural Eastern Ethiopia
Source: Microbiome. 2025 Nov 3;13:225. doi: 10.1186/s40168-025-02203-w (PMC12581456; doi:10.1186/s40168-025-02203-w)

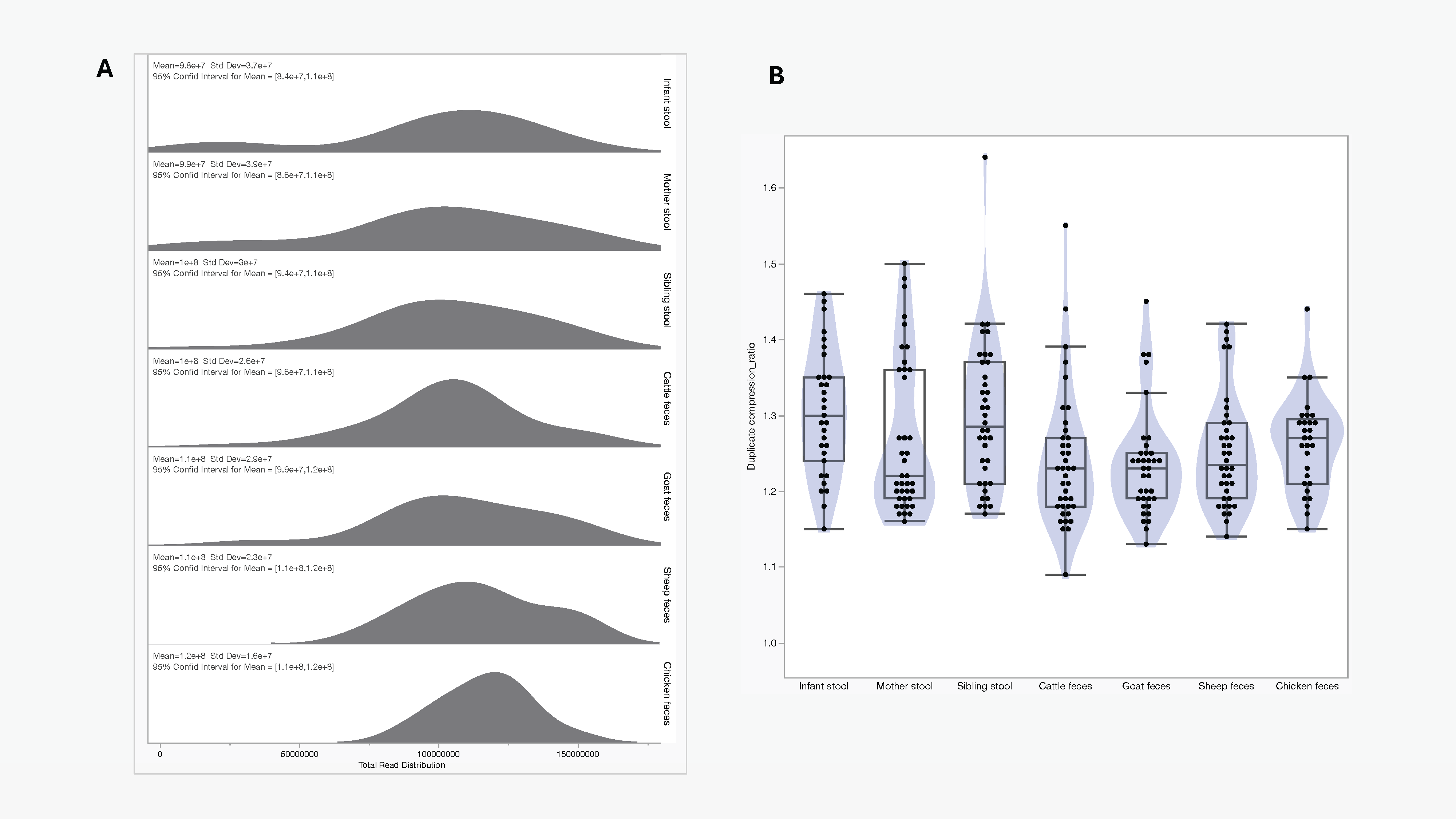

Supplement: Supplementary file 2 — Additional file 1: Fig. S1. Metagenomic sequencing depth and read distribution in seven sample types. A) Density plots showing the distribution of total sequence reads across different sample types. Each curve represents the distribution of sequence read counts for each sample type, with mean values, standard deviations, and 95% confidence intervals (CI). B) The duplicate compression ratio, denoting the ratio of sequences before and after duplicate sequence removal. Values less than 2 conforms the metagenomic sequencing criteria, affirming the absence of sequencing biases. [file 40168_2025_2203_MOESM1_ESM.tiff]

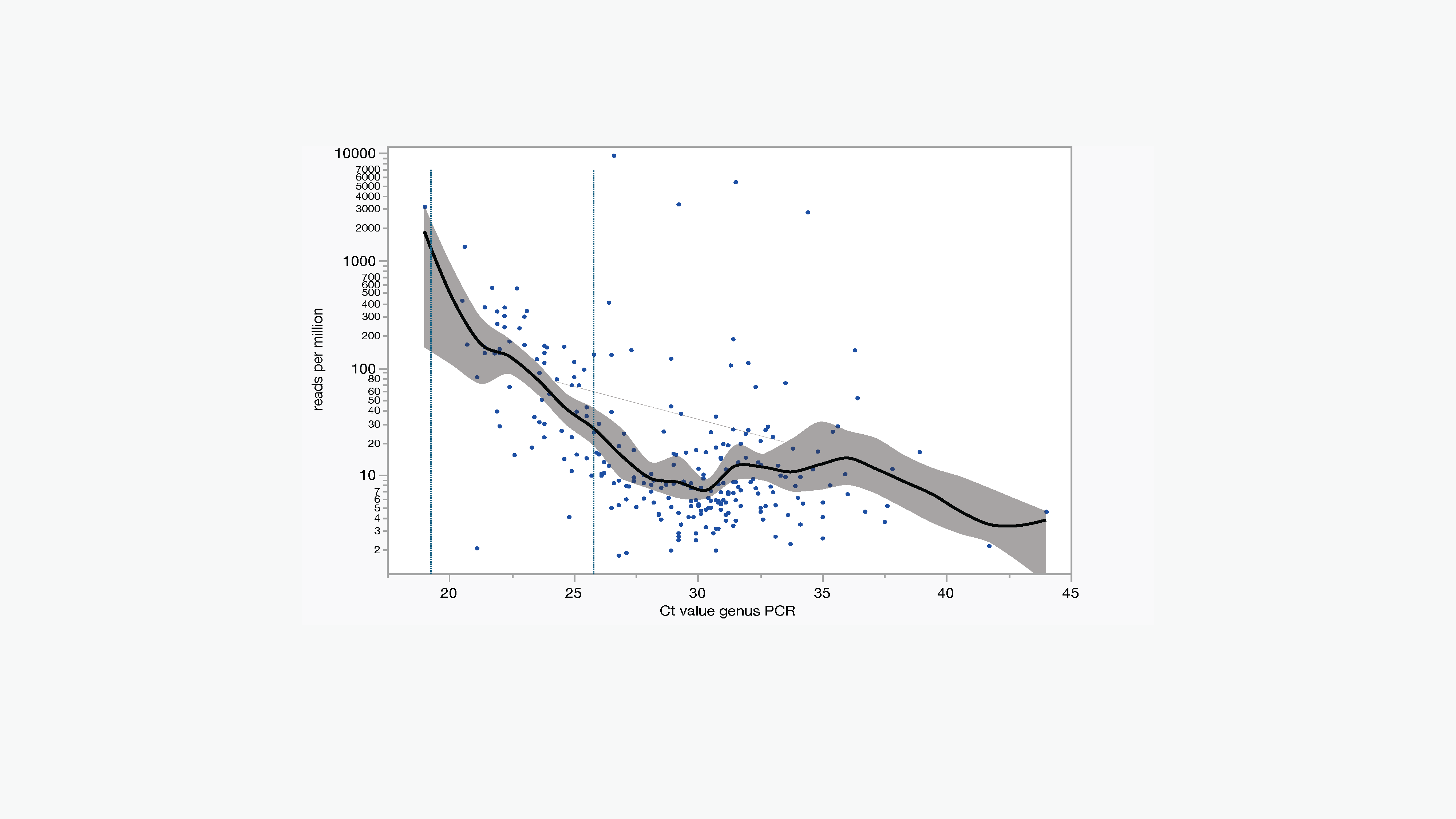

Supplement: Supplementary file 3 — Additional file 2: Fig. S2. Correlation between Campylobacter metagenomic relative abundance and their detection through qPCR. Sliding window analysis depicts the correlation between metagenomic read per million with the genus-level qPCR Ct values. The analysis revealed a Pearson correlation coefficient of -53% (95% CI: -0.68 to -0.31), demonstrating agreement between the metagenomic relative abundance and genuslevel qPCR Ct values. [file 40168_2025_2203_MOESM2_ESM.tiff]

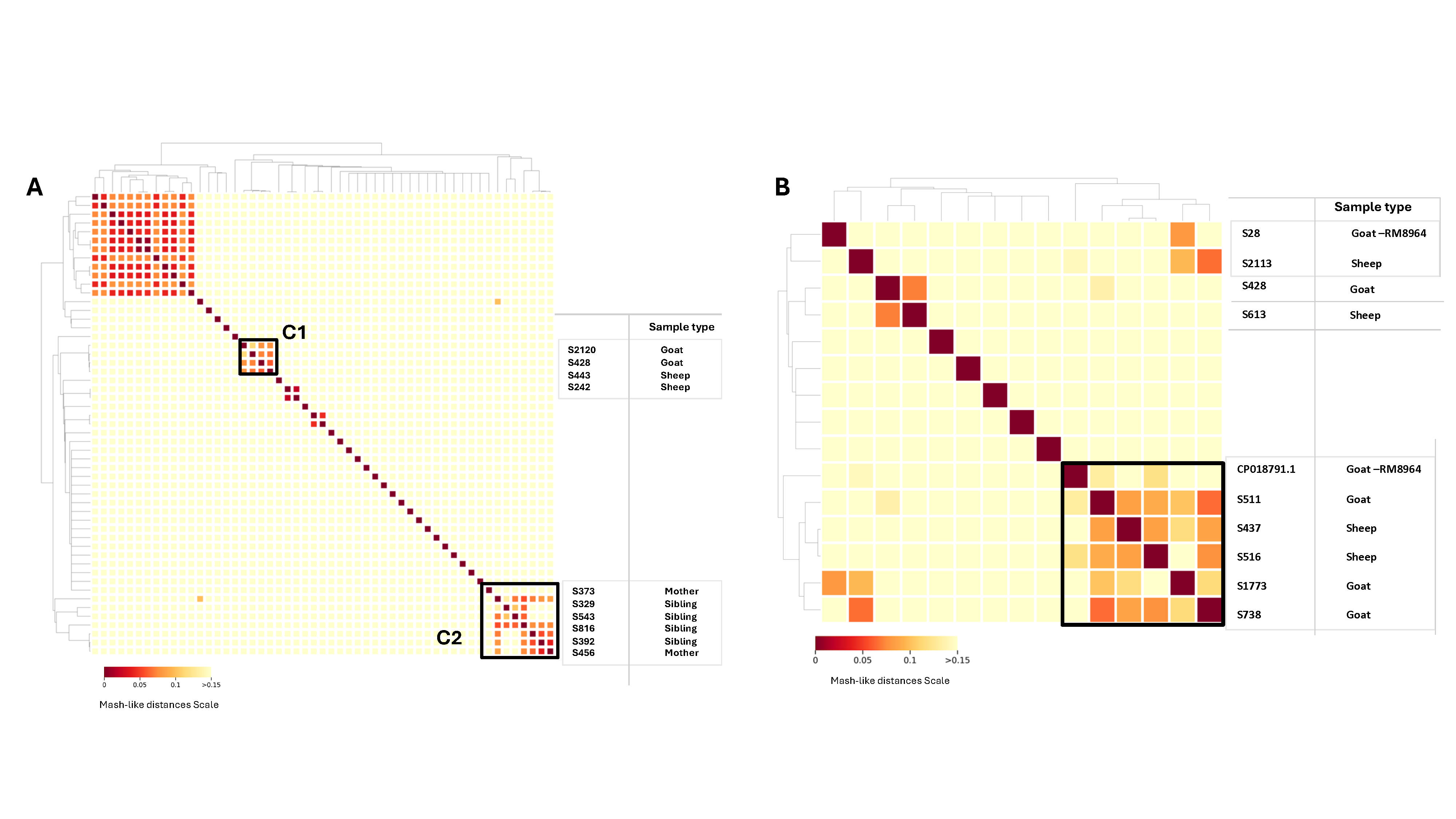

Supplement: Supplementary file 4 — Additional file 3: Fig. S3. Pairwise Mash-like distances for A) Campylobacter Concisus; B) Campylobacter vicugnae. Each sample in the dataset was compared to itself and all other samples. The colors in the matrix represent the range of Mash-like distances (refer to the scale). The diagonal line compares each sample to itself, resulting in dark red squares, indicating zero differences between the sequences. Black boxes are used to show clustering of samples (C1 and C2 in A), and on the right side, sample metadata, including samples and host species are utilized to support the genetic similarities observed in the metagenomic dataset. [file 40168_2025_2203_MOESM3_ESM.tiff]
