# Supplementary material for: Host clustering of Campylobacter species and enteric pathogens in a longitudinal cohort of infants, family members and livestock in rural Eastern Ethiopia
Source: Microbiome. 2025 Nov 3;13:225. doi: 10.1186/s40168-025-02203-w (PMC12581456; doi:10.1186/s40168-025-02203-w)
Supplement: Supplementary file 8 — Additional file 7: S1. The CZ-ID pipeline (Illumina mNGS Pipeline v7.1) was used formetagenomic analysis. [file 40168_2025_2203_MOESM7_ESM.pdf]

**S1. CZ ID Illumina mNGS Pipeline v7.1 pipeline documentation**

**STAR (Implements the step for Host Subtraction)**

--outFilterMultimapNmax 99999
--outFilterScoreMinOverLread 0.5
--outFilterMatchNminOverLread 0.5
--outReadsUnmapped Fastx
--outFilterMismatchNmax 999
--outSAMtype BAM Unsorted
--outSAMmode NoQS
--clip3pNbases 0
--runThreadN {cpus}
--genomeDir {genome_dir}"
--readFilesIn {input files}

**T**rimmomatic (Removes adapter sequences)

java -jar /usr/local/bin/trimmomatic-0.38.jar
PE|SE
-phred33
[input_files]
[output_files]
ILLUMINACLIP:{adapter_fasta}:2:30:10:8:true
MINLEN:35

**Price Seq (**Removes low-quality reads)
PriceSeqFilter
-a 12
-rnf 90
-log c
-fp {input files}
-op {output files}
-rqf 85 0.98

**CZID-dedup (**Identifies duplicate reads.)
czid-dedup
-i {input_fasta}
-o {output_fasta}
-l 70

**Bowtie2 (**Removes remaining host reads.)
bowtie2

-q
-x {genome_basename}
-f
--very-sensitive-local
-S {output_sam_file}
--seed random_seed
-p {number_of_cpus}
-1 [input R1]
-2 [input R2]

GSNAP (remove all human sequences)

gsnapl
-A sam
--batch=0
--use-shared-memory=0
--gmap-mode=all
--npaths=1
--ordered
-t 32
--max-mismatches=40
-D {gsnap_base_dir}
-d {gsnap_index_name}
-o {output_sam_file}
{input_fas}

**SPADES** (short reads are assembled into contigs)

spades.py
-1 {input_fasta}
-2 {input_fasta2}
-o {assembled_dir}
-m {memory}
-t 32
—only-assembler
